# Is It Possible to Improve Working Memory With Prefrontal tDCS? Bridging Currents to Working Memory Models

**DOI:** 10.3389/fpsyg.2020.00939

**Published:** 2020-05-26

**Authors:** Nicola Riccardo Polizzotto, Nithya Ramakrishnan, Raymond Y. Cho

**Affiliations:** ^1^Psychiatry and Behavioral Sciences, University of Texas Health Science Center at Houston, Houston, TX, United States; ^2^Psychiatry and Behavioral Sciences, Baylor College of Medicine, Houston, TX, United States; ^3^Michael E. DeBakey Veterans Affairs Medical Center, Houston, TX, United States; ^4^Menninger Clinic, Houston, TX, United States

**Keywords:** working memory, neurostimulation, TDCS, excitation/inhibition balance, gamma oscillations, computational modeling

## Abstract

A great deal of research has been performed with the promise of improving such critical cognitive functions as working memory (WM), with transcranial direct current stimulation (tDCS), a well-tolerated, inexpensive, easy-to-use intervention. Under the assumption that by delivering currents through electrodes placed in suitable locations on the scalp, it is possible to increase prefrontal cortex excitability and therefore improve WM. A growing number of studies have led to mixed results, leading to the realization that such oversimplified assumptions need revision. Models spanning currents to behavior have been advocated in order to reconcile and inform neurostimulation investigations. We articulate such multilevel exploration to tDCS/WM by briefly reviewing critical aspects at each level of analysis but focusing on the circuit level and how available biophysical WM models could inform tDCS. Indeed, such models should replace vague reference to cortical excitability changes with relevant tDCS net effects affecting neural computation and behavior in a more predictable manner. We will refer to emerging WM models and explore to what extent the general concept of excitation-inhibition (E/I) balance is a meaningful intermediate level of analysis, its relationship with gamma oscillatory activity, and the extent to which it can index tDCS effects. We will highlight some predictions that appear consistent with empirical evidence – such as non-linearities and trait dependency of effects and possibly a preferential effect on WM control functions – as well as limitations that appear related to the dynamical aspects of coding by persistent activity.

## Introduction

The key operations that support an online representation of the world in order to inform context appropriate behavior are collectively referred to as working memory (WM), encompassing both the simple maintenance of information and cognitive control and the adaptive allocation of cognitive resources (Just and Carpenter, 1992; Goldman-Rakic, 1995; Engle et al., 1999; Miller and Cohen, 2001; Vogel and Machizawa, 2004; Chatham and Badre, 2015). Impairments in such functions are critically involved in several disorders, motivating great interest in developing WM boosting interventions. We focus on transcranial direct current stimulation (tDCS), an increasingly popular methodology that brings the promise of a non-invasive, well-tolerated, low-cost, high-yield approach (Bikson et al., 2016; Hill et al., 2016a). Simple rules allowing straightforward predictions also contribute to its appeal. In the context of WM, the standard assumptions follow this simple heuristic: anodal electrode stimulation of the scalp overlying the prefrontal cortex (PFC) will improve WM performance through increases in PFC excitability.

We will briefly review tDCS-WM studies and highlight the mixed findings as well as key mechanistic and theoretical points that could inform reconciling of these mixed results. It is clear that standard assumptions need to be considered as an oversimplification (Bestmann et al., 2015; Jackson et al., 2016). In fact, they fail to explain important aspects of empirical data such as non-linearities, inversions of classical direction effects, polarity neutral effects, state dependency and individual variabilities (Nitsche and Paulus, 2000; Nitsche et al., 2003; Ardolino et al., 2005; Batsikadze et al., 2013; Fresnoza et al., 2014; Esmaeilpour et al., 2018). Moreover, they do not aid the understanding of negative results which likely include many unpublished studies.

The use of multilevel modeling has been advocated to address the complexity of neurostimulation. Mathematical constructs at each level of description – current flow, cell polarization, network and information processing would provide a more informative framework and importantly, suggest relevant neurophysiological correlates. However, there is a remarkable paucity of investigations exploiting such “computational neurostimulation” strategy (Bikson et al., 2012; Bestmann et al., 2015; Rahman et al., 2015; Jackson et al., 2016). Here, we aim at spurring such an approach for WM research with a brief multilevel exploration of the complexity underlying tDCS modulation. The pitfalls of standard assumptions on current models and cellular effects have been extensively reviewed elsewhere (Bikson et al., 2012; Jackson et al., 2016). However, a quantitative detailing of how such lower level effects transfer into relevant WM computations is much less understood. This aspect is of critical importance. Let us assume that all relevant variables, subject and state related, are characterized and incorporated in a protocol that successfully leads to an increase of pyramidal cell excitability or other relevant cellular effects as characterized in animal studies. Why should such changes improve WM? Computational models can start to answer such questions and should be exploited to define the target of stimulation beyond mere gross anatomic considerations. Absent such a framework, the interpretability of empirical findings is limited, and goals and boundaries of stimulation poorly defined. Accordingly, we will elaborate further at this higher-level, highlighting emerging concepts and the appeal of intermediate, network-level dynamics such as prefrontal cortical gamma oscillations with related predictions and inherent shortcomings.

## tDCS and WM Findings

Findings of the effects of tDCS on WM are mixed and a challenge to interpret due to the heterogeneity across multiple factors, all known to potentially affect outcome: tDCS parameters (current direction and density, stimulation duration, number of sessions), protocols (online vs. offline effects, immediate vs. delayed after-effects), individual variability (demographics, genotypes, pathology), subject’s state during stimulation (resting/uncontrolled vs. active/training) and importantly, the WM task used.

A quantitative review (Horvath et al., 2015) evaluated the effects of both online and offline effects on a wide range of cognitive processes, including WM functions in healthy adults and reported overall a null effect. Two analyses (Mancuso et al., 2016; Medina and Cason, 2017) suggest that available evidence is clouded by substantial publication bias with support in favor of real effects being either non-existent (Medina and Cason, 2017) or small and limited to augmentation of training (Mancuso et al., 2016). There is broad consensus that findings from larger homogenous samples is sorely lacking (Brunoni and Vanderhasselt, 2014; Dedoncker et al., 2016; Hill et al., 2016a). However, three other meta-analyses provide support for enhancing effects of anodal-tDCS. Brunoni and Vanderhasselt (2014) and Hill et al. (2016a) showed effects on classic maintenance tests, while Dedoncker et al. (2016) investigated studies addressing both control and maintenance functions.

Indeed, it has been shown that accounting for relevant factors can offer a far more informative perspective than the summary, reductive perspective of metanalysis global outcomes. Hill et al. (2016a) describe an approximately linear relation with current intensity, in agreement with Dedoncker et al. (2016) who further detailed the dependence on current parameters and also showed an interaction with gender. All metanalyses providing positive evidence included a heterogeneous neuropsychiatric population and provide some grounds for positing an interaction between pathophysiology and stimulation protocols, i.e., online vs. offline, and stimulation parameters. Physiological variabilities also play a role in the response. Genetic differences in dopamine metabolism, namely COMT Val(108/158)Met polymorphism affects its critical modulation of PFC function and interacts with tDCS: genotype predicts domain specific online effects on control functions (Plewnia et al., 2013; Nieratschker et al., 2015) and the intensity dependency of longer term effects of WM training paired with tDCS (Stephens et al., 2017).

The extreme variability in WM tasks dictates the need for careful consideration of potential task-dependency in tDCS effects. An intriguing perspective is that effects could be more prominent with increasing control demand. Performance in low difficulty maintenance tasks seems unaffected by tDCS (Berryhill, 2014; Pope et al., 2015), but improvement becomes evident with increasing complexity (Pope et al., 2015) and concomitant control demand (Wu et al., 2014). Performance in these scenarios relies on likely separable cognitive components, yet improvement could arise from enhanced orchestration by cognitive control. While studies specifically addressing cognitive control are sparser, it has been shown that tDCS can improve performance in the paradigmatic cognitive control paradigm, the Stroop test (Ouellet et al., 2015) and two recent studies, not included in any metanalyses showed positive results using variants of the AXCPT, a test believed to provide a purer estimate of control functions (Gómez-Ariza et al., 2017; Boudewyn et al., 2018).

## Current and Cellular Level

It is commonly assumed that changes in excitability derive from polarization of cortical pyramidal cell somas due to radial current flow and that this can be predicted by uniform and localized polarization of the cortex. However, current flow models demonstrate that complex electrical fields arise in biological tissues (Lang et al., 2005; Bikson et al., 2012) and standard bipolar montages involving one electrode placed on the scalp lead to suboptimal PFC involvement (Bikson et al., 2016) with significant inter-subject variability advocating the use of detailed, customized forward models. It follows that characterizing current in terms of the field effectively delivered at target – rather than the use of simple stimulator output intensity – would be a more informative approach allowing consistency across studies, participants and montages, including multielectrode, high resolution methods (Evans et al., 2020).

The effects at the cellular level have been extensively reviewed (Jackson et al., 2016; Cirillo et al., 2017). The assumption of polarity-specific tDCS effects rely on initial animal studies focused on pyramidal cells somas whose firing rate is increased and decreased by inward and outward currents, respectively (Creutzfeldt et al., 1962; Bindman et al., 1964; Purpura and Mcmurtry, 1965; Radman et al., 2009). Human motor cortex studies appear in general agreement as excitability as indexed by transcranial magnetic stimulation (TMS) motor evoked potentials (MEP) appears polarity dependent and increased by anodal stimulation. However, such generalizability needs to be critically re-evaluated (Di Lazzaro and Ziemann, 2013; Rusu et al., 2014) as dendritic and axonal effects together with the involvement of interneurons is supported by most recent animal and modeling studies (Molaee-Ardekani et al., 2013). In addition, this is demonstrated indirectly in humans through magnetic resonance spectroscopy studies (Stagg et al., 2009; Clark et al., 2011; Kim et al., 2014; Hone-Blanchet et al., 2016).

Studies with MEP are fundamental in bridging cellular evidence with pharmacological studies and importantly, in detailing the parameter dependence of effects, including the abovementioned effects that cannot be explained with straightforward linear mapping of polarity and duration. The fact that prefrontal circuitry would respond similarly to the motor cortex could be postulated on the ground of cortical modularity. However, observations are strictly specific to current direction i.e., tangential vs. radial, and sign. Therefore, the relationship between MEP experiments cannot be inferred without including details of prefrontal cortical folding. Direct probes of prefrontal response such has TMS-electroencephalography (EEG) evoked potentials are extremely valuable to this end, yet such approaches offer their unique challenges (Hill et al., 2016b).

At the molecular level the involvement of standard long-term potentiation/depression (LTP/LTD) mechanisms, NMDA receptors and Ca2+-dependent have been consistently described. However, a plethora of other effects affecting AMPA receptors, neurotrophic factors with induction of immediate early genes, modulation by various neurotransmitters and layer specific glia involvement have also been reported. At this level, genetic, pharmacological, and state dependent variables are likely contribute to the consistent inter-individual variability observed in response to neurostimulation. In motor cortex studies up to 50% of subjects do not show MEP modifications following anodal tDCS (López-Alonso et al., 2014; Wiethoff et al., 2014; Strube et al., 2015). Cognitive responses are a less reliable indicator of response and prefrontal stimulation offers other challenges in controlling response variability. A lack of impairment following cathodal stimulation is often observed but such observation does not necessarily question polarity specific reasonings as alternative cognitive strategies can play a compensatory role (Jacobson et al., 2012). However, tDCS molecular targets are affected by activation dependent variables and neuromodulators which vary constitutionally or in reponse to pharmacological interventions. The level of motor cortex activation affects the response to tDCS in MEP studies, yet prefrontal activation is more difficult to define and control (Polanía et al., 2018). Seemingly, motor learning is improved by anodal tDCS but this is conditional on activity-dependent secretion of brain derived neurotrophic factor (Fritsch et al., 2010). In PFC, dopamine plays a critical role in WM and modulates tDCS response both as a function of relevant constitutional differences as mentioned above (Plewnia et al., 2013; Nieratschker et al., 2015; Stephens et al., 2017) and when affected by pharmacological interventions. As reviewed in Stagg and Nitsche (2011), not only dopaminergic agents but also serotoninergic, colinergic, noradrenergic medications, and ions channels blockers could ineract with tDCS and should be controlled in clinical populations (Monte-Silva et al., 2009; Stagg and Nitsche, 2011). The critical importance of careful experiments and outcome measure design for biophysical models has been reviewed in Jackson et al. (2016).

Overall, these studies suggest that the effect on pyramidal neurons can result from a number of different mechanisms that affect both sides of the interacting excitatory (E) and inhibitory (I) cell network. An offset of E/I balance appears to be a more inclusive description of net effects, yet this can arise through a range of cellular and molecular effects which likely vary across the protocols, being online effects related to changes in membrane potential (Nitsche et al., 2003; Stagg and Nitsche, 2011) and offline effects driven by changes in synaptic strength (Liebetanz et al., 2002; Nitsche et al., 2003; Stagg and Nitsche, 2011). Furthermore, excitability or E/I changes do not exhaustively contrast the short-lived effects outlasting stimulation and the longer lasting effects following multiple sessions that are appealing for therapeutic purposes.

## Network and Computation Level

Current, cellular and molecular findings can be incorporated into cortical dynamics models exploiting the sound basis of well-established, biophysically accurate “off the shelf” templates (Durstewitz et al., 2000; Buzsáki and Wang, 2012). Conversely, attempts to reframe assumptions computationally at higher levels are less developed. Furthermore, in spite of growing WM modeling efforts this higher level modeling is not incorporated into tDCS research.

It is assumed that changes in excitability translate directly into effects on cortical operations. Additionally, behavioral changes are predicted, implying that cognitive processes are separable and localized. Yet, what precisely could or should be enhanced by tDCS? Can WM be indeed treated as single dimension separable from other functions? Such fundamental questions can be answered by providing an intermediate level of analysis that bridges the net microcircuitry effects of tDCS with cortical operations. To this end, concepts such as E/I balance, efficiency, zero-sum gain, stochastic resonance, activity-, and input-selectivity have been proposed in order to contrast the simplistic view of a sliding scale effect – i.e., the more excitation the better (reviewed in Bestmann et al., 2015). Such constructs originate from different fields, they are not mutually exclusive and importantly, they cannot be equally translated into physiological measures limiting their heuristic value. From the therapeutic perspective, this resonates with the recent emphasis on measures of target engagement as an essential component in therapeutics development (Insel, 2015). A multilevel perspective indicates that prefrontal cortical engagement needs to be defined beyond anatomy and current, and target cellular dynamics and neurophysiological indices should also be specified.

With the goal of describing a final common pathway of both pathophysiology and meaningful tDCS effects, we will revisit the concept of E/I balance: a basic functional principle underlying cortical dynamics (Isaacson and Scanziani, 2011) which has been also hypothesized as a useful framework for capturing net tDCS effects and its therapeutic potential (Krause et al., 2013). We will refine this general hypothesis and put forward the importance of network level as an appropriate intermediate level of description linking biophysical changes to a relevant physiological output, namely gamma oscillations.

### The Role of E/I Balance

Excitation/inhibition balance refers to the relative contributions of excitatory and inhibitory synaptic inputs to some neuronal event and are said to be balanced if their ratio is constant across a wide range of conditions. Such E/I homeostasis is described as an ubiquitous phenomenon most extensively documented in the neocortex including PFC (Yizhar et al., 2011). It is believed to serve as a major gain mechanism that optimize neural coding, information propagation (Zhou et al., 2014) and plasticity (Cohen-Kashi Malina et al., 2013) extending the appeal of E/I balance as an indicator of tDCS net effects. Krause et al. (2013) put forward such a perspective and advocated for ratios of glutamate/GABA as a simple yet useful target of modulation. It is hypothesized that E/I represents a meaningful unidimensional representation of tDCS effects where final outcome depends also by the initial position which can vary across individuals which could be indexed *in vivo* by magnetic resonance spectroscopy glutamate/GABA signatures. Indeed variability in E/I is described to be relevant to both physiological differences (Jocham et al., 2012) and various neuropsychiatric disorders such as schizophrenia, autism, ADHD (Krause et al., 2013; Kuo et al., 2014; Hill et al., 2016a).

It should be emphasized that the privileged role of the E/I balance concept stems from a generalization of a plethora of observations, each accurately defined in terms of spatiotemporal details, where glutamate/GABA provides only a partial and static picture. For instance, excitatory inputs can be further described in terms of NMDA vs. AMPA dynamics, which are differently involved in tDCS mechanisms. The inhibitory drive stems from a great cellular diversity in GABAergic interneurons, offering distinctive behaviors in presence of an electric field (Huang and Paul, 2019). The major source of inhibitory input is provided by parvalbumin positive cells which are involved in many well documented instances of E/I balance (Atallah et al., 2012; Moore and Wehr, 2013; Zhou et al., 2014). However, the role of other classes has also been suggested (Atallah et al., 2012; Lee et al., 2012; Wilson et al., 2012). Considering temporal dynamics, *transient* E/I balance is described as underlying fast neuronal events and adaptations while population differences refer to the so stated *global* E/I balance (Okun and Lampl, 2009). Clearly, the latter also plays a dynamic role, but this occurs as a function of the kinetics of the actual cellular and molecular underpinnings. It is therefore critical to contextualize E/I balance with models specific to WM.

A large body of evidence points to the importance of E/I balance in WM, which has been integrated in computational models. Murray and Wang (2018) detailed how perturbing the conductance strengths of excitatory currents onto pyramidal cells vs. interneurons could upset E/I balance and spatial WM representations. Lim and Goldman (2013) emphasized the role of persistent activity and put forward how robust representations could be achieved by recurrent excitatory and inhibitory inputs balanced in strength and offset in time, implementing a mechanism akin to a corrective negative feedback. On the time scale dictated by the decay constant of inhibitory GABA-mediated currents, fast interactions of E/I cells are related to circuit level oscillations in the gamma frequency (30–80 Hz, Vierling-Claassen et al., 2010; Buzsáki and Wang, 2012). Prefrontal cortex gamma oscillations are strongly associated with WM functions, as most clearly demonstrated by intracranial recordings (Bahramisharif et al., 2018; Bartoli et al., 2018). Indeed, the integrity of the rhythmogenic pyramidal-interneuron network is essential for WM (Sawaguchi et al., 1989; Whittington et al., 1995; Traub et al., 1996; Gonzalez-Burgos and Lewis, 2008). Consistently, WM impairment in conditions such as schizophrenia is associated with weakened gamma activity in both maintenance (Uhlhaas and Singer, 2010) and control tasks (Cho et al., 2006; Minzenberg et al., 2010).

Modeling work points to dissociable effects of excitatory conductances. Murray and Wang (Murray and Wang, 2018) provided an account for a role of specific disturbances in NMDA conductance for excitatory synapses onto interneurons consistent with alterations associated with WM impairment in schizophrenia. Wang (Wang, 2006) also noted the separate contributions of AMPA vs. NMDA strengths in sustaining gamma activity where the fast-kinetics of AMPA allow for the quick cycling through the on-off of each oscillation and the slower kinetics of NMDA support continued network activation from one cycle to the next.

Such computational models also help in lending precision to dose/response curves and therapeutic goals. For instance, in Figure 1 from Murray and Wang (2018), we see the extent to which E/I balance modulates the precision of spatial WM representations. The spatial working memory task involved presentation of a spatial cue after which a representation of that spatial location was maintained over a delay period. Sufficient mutual excitation (G_EE_, the conductance strength between pyramidal neurons) of local pyramidal neurons was necessary to sustain persistent activity representing that location across the delay period. Sufficient excitation of inhibitory neurons (G_EI_, the conductance strength of excitation from pyramidal onto inhibitory neurons) was necessary to inhibit activity of neurons representing irrelevant locations. The critical feature is the existence of iso-contour lines along the positive slope directions, representing stable E/I ratios and equivalent spatial WM performance despite changes in the absolute conductance values. Such a “sloppy” axis defines a robust balance that contrasts with a “stiff” axis over which even modest E/I balance changes results in striking changes in representations. Poor WM performance arises either both from a relative defect of excitation by pyramidal cells onto other pyramidal cells (unstable persistent state; upper left of plot) or onto inhibitory interneurons, resulting in widespread excitation (unstable baseline state; lower right of plot). Thus, with poor performance at either extreme of this “stiff” axis, we see WM performance can be a non-linear, inverted-U function of E/I balance.

**FIGURE 1:**
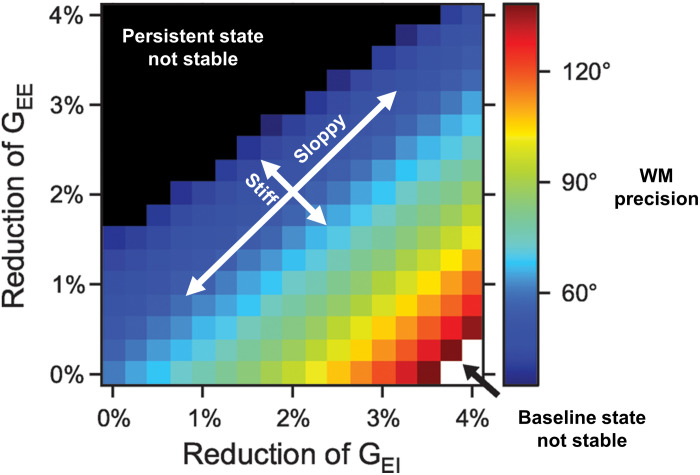
Working memory (WM) precision as a function of excitation/inhibition balance (adapted from Murray and Wang, 2018). NMDAR conductance strengths onto pyramidal (G_EE_) and inhibitory cells (G_EI_) are depicted along the y- and x-axes, respectively. Iso-contour lines span positive slope directions, along which E/I ratios are associated with equivalent spatial WM precision despite changes in the absolute conductances. Optimal performance results in the region around the diagonal. Such a “sloppy” axis defines a regime where significant changes in conductance parameters values still preserve E/I ratios and WM performance. This contrasts with a “stiff” axis over which even modest E/I balance changes result in significant changes in WM performance. Along this stiff axis, poor WM performance arises from one of two ways: a relative defect of excitation by pyramidal cells onto other pyramidal cells (unstable persistent state; upper left of plot) or onto inhibitory interneurons, resulting in widespread excitation (unstable baseline state translating to representations of all spatial locations being indiscriminately activated; lower right of plot). Thus, with poor performance at either extreme of this “stiff” axis, we see WM performance can be a non-linear, inverted-U function of E/I balance.

Accordingly, insofar as tDCS affects E/I and that the biological variability spans across the explored parameter space, the effects on WM are expected to be non-linear and – in alignment with the above mentioned general E/I balance reasoning – the magnitude and direction of effects depends on individual pre-stimulation values. We can then note three theoretical main implications. First, since an optimal value exists – which is assumed to fall within the physiological range corresponding to normal or high performance – WM can be improved by tDCS if parameters are individually adapted. However, the second implication is that effects have an upper limit. The optimal level corresponds to subjects that would not benefit from tDCS of either polarity or any duration. On the contrary, their performance could actually deteriorate with any tDCS. Lastly, absolute excitatory and inhibitory values are immaterial and tDCS can restore balance across conditions regardless of the actual circuit disturbances. In the context of the NMDA hypofunction hypothesis of schizophrenia, this would point to the possibility of improving WM through balancing E/I by tuning tDCS parameters in order to shift from the lower right of the plot to a point along the diagonal. The proposed model does not address directly oscillatory dynamics. However, similar non-linear, invert-U relationships have also been observed for network gamma oscillations in computational models that vary the excitability of parvalbumin positive interneurons (Kömek et al., 2012; Komek et al., 2014). Accordingly, the fast kinetics E/I balance modeled as pyramidal-interneuronal gamma emerges as a meaningful level of analysis of tDCS effects on WM which also offers neurophysiological measures of target engagement, i.e., prefrontal gamma.

Such theoretical perspective clearly emphasizes the practical importance to measure E/I *in vivo* in order to define pre-stimulation individual values. We addressed how glutamate/GABA indices such as magnetic resonance spectroscopy measures can only be considered as a proxy and do not provide the spatiotemporal details relevant to modeling. However, the same connection that relates prefrontal gamma to E/I – with related strengths and limitations – can be exploited to this end. Measuring cortical activity during WM tasks would appear to be a straightforward approach as it defines the very target of remediation by tDCS. However, such measures are confounded by differences in performance and execution strategy. Such hurdle can be overcome by using TMS-EEG evoked responses in the spectral domain (Hill et al., 2016b) in order to tailor tDCS at the single subject level.

An inverted-U dependency has also been shown between WM and dopamine as demonstrated in with pharmacological modulation with dopaminergic agents (Vijayraghavan et al., 2007) and by relevant constitutional differences (COMT Val(108/158)Met polymorphism; Cools and D’Esposito, 2011). Seemingly the noted interaction between tDCS effects and dopaminergic system is consistent with such a non-linearity (Monte-Silva et al., 2009; Plewnia et al., 2013; Nieratschker et al., 2015; Stephens et al., 2017). Together, these findings suggest that cortical function and WM performance have a multi-parametric non-linear dependency on dopamine and E/I, with such complex dependencies likely extending to other physiologic and psychological parameters. Empirical sampling of such a multidimensional parameter space will yield important information. However, capturing such complexity will likely require the detailed mechanistic framework of computational models also capable of addressing the relation between factors like dopamine and E/I. For instance, Kömek et al. (2012) paired modeling work with the empirical finding of a differential effect of a single dose of amphetamine on cortical gamma in schizophrenia patients and healthy controls. Results were consistent with an inverted-U function where an increase in dopaminergic output improved gamma oscillations in patients, believed to start from an hypodopaminergic state, but had a detrimental effect in controls which would transition into a suboptimal hyperdopaminergic state. Such observation was successfully captured through simulations that modeled dopamine effects on the E/I balance through modulating fast-spiking interneuron excitability. While direct modeling is required, one synthetic hypothesis would be that the dopaminergic level is one of the factors that set the initial position in the E/I space and predicts tDCS effects accordingly.

### Persistent Activity and Alternative Mechanisms

The appeal of the E/I framework is intertwined with the role assigned to persistent activity and sustained gamma in WM and needs to be confronted by observations that suggests the contribution of other mechanisms and differences between WM functions. Prevailing models support the critical role of active patterns in PFC and oscillatory mechanisms including interactions between gamma and lower frequencies, namely cross frequency coupling, which have been shown to explain some control functions (Bahramisharif et al., 2018; Miller et al., 2018) so that representations can be protected from interference and cleared (Dipoppa and Gutkin, 2013; Dipoppa et al., 2016). Interestingly, models that include dynamic aspects like binding and multiple representations have highlighted the role of mutual inhibition between subpopulations (Pina et al., 2018). Indeed, a growing understanding of the dynamic aspects of persistent activity uncovers the existence of distinct, complementary mechanisms. The stability of neural representations called WM emerges as a population level configuration from underlying organized instability. The subpopulation of neurons coding for task variables and even neuronal selectivity are believed to be time varying (Murray et al., 2017b; Spaak et al., 2017). This is consistent with observations of activity being organized as discrete, non-continuous oscillatory events and spiking (Lundqvist et al., 2016) where individual neuron involvement depends on its intrinsic temporal properties (Wasmuht et al., 2018).

The amount of recruited persistent activity is task-dependent, which has been explained by models suggesting that information can be maintained in an activity-silent fashion by short-term synaptic plasticity (STSP, Zucker and Regehr, 2002; Mongillo et al., 2008; Masse et al., 2018). Consistently, empirical evidence shows that representations can be reactivated by probing a silent circuit (Rose et al., 2016; Wolff et al., 2017). However, most recently Masse at al. (2018) also showed that STSP alone cannot sustain some control-related WM functions where persistent activity is necessary and its strength increases with the degree of the required control.

The dynamic perspective on persistent activity does not necessarily diminish the appeal of the fast-acting E/I balance framework to inform tDCS/WM. Indeed, the use of non-invasive stimulation methods as transcranial alternating current stimulation (tACS) to probe the causal role of oscillatory activity support the involvement of appropriately timed gamma oscillations (reviewed in Albouy et al., 2018). Overall, models suggest that a positive effect of tDCS can be more reliably predicted on control functions, as the existence of other mechanisms sustaining maintenance exposes the response to other sources of variability. As noted above, this observation could be consistent with available empirical evidence. However, suboptimal prefrontal E/I accounts only for a portion of the individual variability in WM. Other factors are either independent or unclearly related to this possible tDCS net effect.

Both local microcircuit connections and long-range connectivity are critically involved in WM. Murray et al. (2017a) showed that despite important temporal aspects to neural dynamics, population-level WM representations in PFC are stable when certain local connectivity properties are implemented. Biophysical multiregional models are far less investigated. However another model, Murray et al. (2017b) captured the interaction between PFC and the posterior parietal cortex which is at the core of the network of brain areas underlying WM operations. Such work suggests a distinctive role for PFC persistent activity. While parietal areas transiently encode distractors and accumulate evidence supporting target selection, PFC preserves a robust representation of targets by filtering distractors and organizing categorical selections.

The dynamic aspects of persistent activity and the involvement of other mechanisms makes tDCS effects less easily predictable. For instance, the basis of STSP – which is distinct from the more commonly investigated LTD/LTP – should be addressed. Similarly, tDCS could influence the mutual inhibition between subpopulations underlying multiple representations (Pina et al., 2018). It follows that tDCS could have positive effects, additive or synergistic to the impact on persistent activity, but it could also have antagonistic effects leading to trade-offs. Modeling by Pereira and Wang (2015) showed that NMDA dependent recurrent excitation is critical to the maintaining persistent activity, whereas slow synaptic or cellular processes contribute to the robustness of representations leading to an accuracy-flexibility tradeoff. This is physiologically adjusted to behavioral demands but could be negatively affected by tDCS. Correlational evidence and tACS evidence emphasizes the importance of cross frequency coupling (Albouy et al., 2018). Furthermore recent empirical evidence suggests that tDCS effects on WM can indeed be accompanied by modulation of nesting of gamma cycles within slower theta rhythms (Jones et al., 2020). However, there are no models clarifying mechanistically the impact on coupling and its relation to E/I. Models of local connectivity (Murray et al., 2017a) suggest that the efficiency of population coding is unlikely to be affected short term by tDCS short term, while long term effects could be possible only through mechanisms that cannot be easily be captured by the E/I framework alone, for instance involving growth factor release. Models of the interaction between PFC and parietal areas (Murray et al., 2017b) not only predicts dissociable effects of affecting E/I in the two regions, but clearly stresses the synergistic nature of their action that can be altered in ways that are still unpredictable. It should be noted that imaging studies provide some evidence of long-range functional connectivity changes following tDCS that, in some instances, are related to behavioral effects (Wörsching et al., 2016). However, the reliability of these findings is unclear (Worsching et al., 2017) and there are no explicit efforts to relate such changes to E/I or to biophysical models in general.

These arguments start addressing the question of whether WM modulation can be captured along a single dimension and whether it is possible to target WM in isolation. It is unclear if the E/I balance perspective provides basis for an effect that would generalize equally to all prefrontal operations as might be expected by a basic optimization principle underlying cortical dynamics. Overlooking functions regarded as dissociable, i.e., maintenance vs. control or even antagonistic such as sustained representation vs. flexible update (Goschke, 2000; Armbruster-Genç et al., 2016) needs to be regarded as further oversimplifications. Different cognitive processes may themselves pose as constraints to each other, where an optimal, dynamic titration across competing task demands necessarily imposes tradeoffs as it has been demonstrated for tDCS in other domains (Iuculano and Cohen Kadosh, 2013).

## Summary and Conclusion

Empirical studies suggest that prefrontal tDCS can improve WM under certain conditions, with effects being possibly more prominent with increasing control demand. However, the most salient aspect of available findings seems to be the variability of response. At each level of description, we have outlined some of the existing uncertainties, corresponding sources of variability and shortcomings of standard assumptions.

Our overview is grounded in two themes of general relevance to neurostimulation. First, a multilevel perspective indicates that anatomical precision cannot be meaningfully exploited unless targets are also defined in terms of computationally relevant changes to cellular dynamics. Second, while our understanding appears to decrease moving up from the level of current flow, the gap from cellular effects to behavior can also rely on increasingly sophisticated quantitative approaches. Biophysically accurate, domain specific models allow answering basic questions like what exactly is affected by stimulation and why it should lead to behavioral changes.

In tackling the impact of tDCS on WM, the weakness of anatomical precision is further complicated by the necessity to rely on forward models of current in order to target PFC. The overlying scalp does not have the special role played in TMS and tDCS is anatomically imprecise unless montages are guided by detailed, individualized models. Basic findings from cellular and MEP studies cannot be transferred to WM enhancement unless anatomical precision include the relative geometry of electrical fields and cortical folding. Appropriate neurophysiological methods such as TMS/EEG allow direct indexing of prefrontal effects and can assist in deriving regional response curves beyond simple modular reasoning.

Emerging WM models provide some understanding of empirical findings insofar WM may be supported by persistent activity. The general E/I balance framework promises to capture mechanistic relevant net tDCS effects but lacks the necessary spatiotemporally resolution. WM models help to overcome such limitations and highlight the existence of an optimal E/I balance where identical performance can be achieved regardless of absolute excitatory and inhibitory values. Accordingly, effects should be non-linear, and their magnitude and direction depend on individual pre-stimulation values. Therefore, improvement requires customization of parameters with an upper limit represented by E/I optimization. To the extent that WM impairments are related to E/I imbalance, tDCS could restore balance regardless of the actual circuit disturbances supporting tDCS use in various disorders where E/I imbalance has been postulated.

Oscillatory activity and fast kinetic E/I balance underlying prefrontal gamma represents a meaningful level of analysis, which also offers a neurophysiological measure of target engagement and meaningful pre-stimulation individual characteristic. Furthermore, it provides the cellular and molecular resolution needed to contextualize tDCS effects: online, immediate and prolonged after effects are likely to affect the rhythmogenic pyramidal-interneuron network in different ways and the outcome cannot be predicted at the general E/I balance level.

A WM improvement can be expected when active representations are suboptimal due to fast kinetic E/I imbalance. However, a growing understanding of the dynamic aspects of representations highlights the importance of other mechanisms that are poorly addressed by tDCS cellular studies and modeling. Effects on control functions can be more reliably predicted as recent evidence suggests that persistent activity is necessary for control functions while maintenance could rely on silent representations. Furthermore, additional mechanisms are expected when multiple representations are at play and the relation with other WM underpinning such as cross frequency coupling and connectivity awaits to be formally addressed. Clearly, there is no *a priori* reason why tDCS effects should be limited to the machinery underlying active representations. Additional changes could occur in either direction leading to either additive/synergistic or antagonistic effects with related trade-offs.

Multilevel modeling offers a space where tDCS parameters under our control can be effectively tracked. Such a parameter space is extremely vast, spanning across stimulation parameters that include intensity, duration, and number of sessions, as well as montages, including high resolution setups. Yet models can define upper boundaries and incorporate safety and tolerability considerations in defining the limits to what can be accomplished *in vivo*. Furthermore, such perspective motivates the integration of relevant single subject specific information from different methodologies into a tailored approach capable of controlling for and exploiting relevant sources of variability. This includes not only the morphological and connectivity details derived from structural and functional MRI methods and EEG (Sanchez-Todo et al., 2018), but also individual pre-stimulation values on dimensions of interest at higher level like E/I with neurophysiological approaches such as TMS/EEG.

In the attempt to provide a general outline encouraging a quantitative, multilevel approach in tDCS/WM, our overview has limitations. We pointed to available thorough reviews of the present understanding of the current and cellular level (Jackson et al., 2016; Cirillo et al., 2017). In addressing one way to capture the transfer of cellular and molecular net effects to models relevant to behavior, we left out competing or complementary frameworks. Furthermore, by simplifying WM into maintenance and control functions we neither addressed the complexity of cognitive constructs related to prefrontal operations, nor the fact that such operations pertain to a node in a broader network sustaining behavior. However, the goal was emphasizing that even such questions could be addressed in a quantitative framework as models allow perturbations and measurements inaccessible to *in vivo* experiments. The Holy Grail is identifying a unifying transfer function that, if positively affected, would improve fundamental prefrontal computation and generalize to all subserved functions. It is unclear if the E/I balance perspective satisfies this requirement, but this is a testable hypothesis.

## Author Contributions

NP wrote the first draft of the manuscript. NP, NR, and RC wrote sections of the manuscript. All authors contributed to manuscript revision, read and approved the submitted version.

## Conflict of Interest

The authors declare that the research was conducted in the absence of any commercial or financial relationships that could be construed as a potential conflict of interest.
